# No Significant Increase in the Δ4- and Δ7-Dafachronic Acid Concentration in the Long-Lived *glp-1* Mutant, nor in the Mutants Defective in Dauer Formation

**DOI:** 10.1534/g3.115.018812

**Published:** 2015-05-12

**Authors:** Tie-Mei Li, Weilong Liu, Shan Lu, Yan-Ping Zhang, Le-Mei Jia, Jie Chen, Xiangke Li, Xiaoguang Lei, Meng-Qiu Dong

**Affiliations:** * National Institute of Biological Sciences, Beijing, Beijing 102206, China; † Beijing National Laboratory for Molecular Sciences, Key Laboratory of Bioorganic Chemistry and Molecular Engineering of Ministry of Education, Department of Chemical Biology, College of Chemistry and Molecular Engineering, Synthetic and Functional Biomolecules Center, and Peking-Tsinghua Center for Life Sciences, Peking University, Beijing 100871, China

**Keywords:** dafachronic acid, steroid hormone, *glp-1*, *daf-12*, *C. elegans* dauer

## Abstract

The steroid hormone dafachronic acid (DA) regulates dauer formation and lifespan in *Caenorhabditis elegans* by binding to the nuclear receptor DAF-12. However, little is known about how DA concentrations change under various physiologic conditions and about how DA/DAF-12 signaling interacts with other signaling pathways that also regulate dauer formation and lifespan. Using a sensitive bioanalytical method, we quantified the endogenous DA concentrations in a long-lived germline-less *glp-1* mutant and in the Dauer formation-defective (Daf-d) mutants *daf-12*, *daf-16*, *daf-5*, and *daf-3*. We found that the DA concentration in the *glp-1* mutant was similar to that in the wild type (WT). This result is contrary to the long-held belief that germline loss-induced longevity involves increased DA production and suggests instead that this type of longevity involves an enhanced response to DA. We also found evidence suggesting that increased DA sensitivity underlies lifespan extension triggered by exogenous DA. At the L2/L3 stage, the DA concentration in a *daf-12* null mutant decreased to 22% of the WT level. This finding is consistent with the previously proposed positive feedback regulation between DAF-12 and DA production. Surprisingly, the DA concentrations in the *daf-16*, *daf-5*, and *daf-3* mutants were only 19–34% of the WT level at the L2/L3 stage, slightly greater than those in the Dauer formation-constitutive (Daf-c) mutants at the pre-dauer stage (4–15% of the WT L2 control). Our experimental evidence suggested that the positive feedback between DA and DAF-12 was partially induced in the three Daf-d mutants.

In *Caenorhabditis elegans*, the steroid hormone dafachronic acid (DA) is known to regulate both development and lifespan (Antebi 2013; Sommer and Ogawa 2011). Under standard culture conditions, *C. elegans* undergo four larval stages (L1−L4) after hatching, and develop into adults with an average lifespan of 3 wk. The term ‘dauer’ refers to a special developmental stage alternative to the normal third larval stage (L3); it is a diapause state that is normally induced by harsh conditions (Cassada and Russell 1975). DA exerts its function by binding to the nuclear hormone receptor encoded by *daf-12*, which was initially identified as a common target downstream of several signaling pathways that regulate dauer formation, including insulin signaling and transforming growth factor beta (TGF-β) signaling (Fielenbach and Antebi 2008; Rottiers and Antebi 2006; Thomas *et al.* 1993; Vowels and Thomas 1992). Mutations that inactivate the insulin receptor gene *daf-2* or the TGF-β gene *daf-7* cause *C. elegans* to arrest as dauer larvae during development; this is called the Daf-c (Dauer formation-constitutive) phenotype. The Daf-c phenotype of *daf-2* mutants can be suppressed by mutations in the downstream transcription factor gene *daf-16* (Fielenbach and Antebi 2008). Similarly, the Daf-c phenotype of *daf-7* mutants can be suppressed by mutations in either of the two downstream transcription factor genes *daf-5* or *daf-3* (Fielenbach and Antebi 2008). The daf*-16*, *daf-5*, and *daf-3* mutants are all defective in dauer formation, a phenotype referred to as Daf-d.

As mentioned, DA is also involved in lifespan regulation (Rottiers and Antebi 2006). Ablation of the germ-cell precursors in *C. elegans* increases lifespan in a *daf-12−* and *daf-16*−dependent manner (Hsin and Kenyon 1999). The lifespan extension induced by germline loss requires an intact somatic gonad and is generally believed to involve increased DA production (Kenyon 2010). For example, the *glp-1(e2141ts)* mutant develops a gonad with no germ cells at the restrictive temperature of 25° and lives longer than the wild type (WT) (Arantes-Oliveira *et al.* 2002). Worms lacking the entire gonad (germ cells and somatic cells) did not live longer than intact animals, and their lifespan could be extended by the application of exogenous DA (Yamawaki *et al.* 2010). Recently, it has been reported that there is a fivefold increase of DA in the *glp-1(e2141)* mutant, relative to WT, at 25°, and that increased DA/DAF-12 signaling up-regulates the expression of the *let-7* family miRNAs, which are negative regulators of *akt-1*. The protein product of *akt-1* is responsible for transmitting the inhibitory signal from DAF-2 to DAF-16 (Shen *et al.* 2012).

Although DA/DAF-12 signaling has critically important biological functions, little is known about the physiological concentrations of DA, because of technical difficulties in DA measurement. We recently developed an accurate and sensitive liquid chromatography-mass spectrometry (LC-MS) method to quantify the endogenous DA concentration in *C. elegans* larvae and adults (Li *et al.* 2013). We used this method previously to quantify the DA levels in Daf-c mutants at the pre-dauer stage and found that they are indeed DA deficient. In this study, we measured DA concentrations in Daf-d mutants and in the long-lived *glp-1* mutant.

## Materials and Methods

### Worm strains and chemicals

The WT (Bristol N2), *daf-16(mu86)*, *daf-12(rh61rh412)*, *hsf-1(sy441)*, *glp-1(e2141)*, *glp-1(e2144)*, *gon-2(q388)*, *daf-3(mgDf90)*, *daf-5(e1386)*, and *daf-16*::*gfp(zIs356)* strains were provided by the Caenorhabditis Genetics Center. Transgenic strains expressing an HA-tagged DAF-12 or a GFP-tagged DAF-16, or both: MQD500 *hqEx99 [pLS2 (Phsp-16.2*::*HA*::*daf-12b*::*FLAG*::*His)*; *pCFJ90 (Pmyo-2*::*mCherry)*; *pRG5271(NeoR)]*, MQD 690 *hqEx243[pDYH5 (Pdaf-16*::*daf-16*::*His*::*GFP)*; *pCFJ90 (Pmyo-2*::*mCherry)*; *pRG5271(NeoR)]*, and MQD 687 *hqEx240 [pLS2 (Phsp-16.2*::*HA*::*daf-12b*::*FLAG*::*His)*; *pDYH5 (Pdaf-16*::*daf-16*::*His*::*GFP)*; *pCFJ90 (Pmyo-2*::*mCherry)*; *pRG5271(NeoR)]*.

[5, 24, 25-D3]-25*S*-Δ7-DA was synthesized in-house (Liu *et al.* 2015). 25*S*-Δ7-DA was obtained from two sources: provided by Dr. Adam Antebi (Max Planck Institute for Biology of Ageing, Cologne, Germany) (Motola *et al.* 2006) or synthesized in-house (Liu *et al.* 2015). 25*S*-Δ7-DA was provided by Dr. Hans-Joachim Knölker (Technische Universität Dresden, Germany) (Martin *et al.* 2008)

### Worm culture

To obtain synchronized worm cultures for the LC-MS and real-time polymerase chain reaction (PCR) experiments, eggs were obtained by bleaching gravid adults. After hatching, synchronized L1 larvae were placed onto high-growth plates seeded with *Escherichia coli* OP50 and cultured at 20 or 25°. The developmental stages were confirmed by examining the worms under a microscope before harvest. The *glp-1(e2141)*, *glp-1(e2144)*, *gon-2(q388)*, and N2 control worms were harvested at three developmental stages: L2 larvae, L3 larvae, and day-1 adults. The Daf-d mutant worms were harvested during the transition period between the L2 and L3 larval stages. Three (for *glp-1(e2141)* and the Daf-d mutants) or six (for *glp-1(e2144)*) independent cultures were harvested and examined in these experiments.

### LC-MS quantification of DA

LC-MS quantification of DA was performed as described previously (Li *et al.* 2013). To summarize, internal standard [d3]-DA was added to 150 μL of worm pellets, followed by homogenization and extraction of total lipids with chloroform/methanol (2:1). The total lipid fraction extracts were dried under nitrogen gas and derivatized by successively adding 100 μL of triphenylphosphine (10 mM in acetonitrile), 100 μL of 2,2´-dipyridyl disulfide (10 mM in acetonitrile), and 100 μg of 2-picolylamine (in 100 μL of acetonitrile), and incubating the final mixture at 60° for 20 min. The derivatization reaction was quenched by the addition of 100 μL of methanol/acetic acid (99:1). The derivatized products were dried under nitrogen gas and reconstituted in 60% acetonitrile before LC-MS analysis. Samples were loaded on a C18 column (Hypersil Gold column, 50 mm long, 1 mm i.d., 3-μm particle size; Thermo Fisher Scientific) using a Suveyor autosampler (Thermo Fisher Scientific). DA and [d3]-DA were analyzed with a Q Exactive hybrid quadrupole-Orbitrap mass spectrometer (Thermo Fisher Scientific) in the selected ion monitoring mode (resolution = 70,000). Peaks corresponding to DA and [d3]-DA were extracted with a mass tolerance of 10 ppm, and the peak area was calculated using Xcalibar software version 2.2 SP1.48 (Thermo Fisher Scientific). Each derivatized sample was analyzed three times (three technical repeats). The amount of DA in each sample was averaged over the three technical repeats and normalized to the total protein amount.

### Lifespan assay

Lifespan assays were carried out at 20, 25, or 28°. DA was mixed with *E. coli* OP50 and seeded on the plates several hours before use. The final DA concentrations were: Δ4-DA, 400 nM; Δ7-DA, 200 nM. Ethanol was used as a control. To synchronize worms, 20 adult worms were allowed to lay eggs on NGM plates for 4 hr before being removed. The progeny were transferred to new plates containing DA or ethanol (10 worms/plate) when they grew to adult day 1. At least 10 plates (100 worms) were used in the lifespan assay for each treatment. Worms were transferred to fresh plates every day until they ceased laying eggs, after which worms were transferred to fresh plates once each week. Kaplan-Meier survival analysis was performed with SPSS software and the *p* values were calculated using the log-rank method.

### DAF-16 localization assay

Transgenic worms expressing DAF-16::GFP were shifted from 20° to 25° or 28° on adult day 1. After 12 hr, the localization of DAF-16::GFP was examined.

### Quantitative reverse-transcription PCR

Total RNA was extracted from synchronized worms using TRIZOL (Invitrogen), followed by the removal of contaminant DNA using DNase I. For real-time, quantitative PCR (qPCR) of mRNA levels, template complementary DNA was synthesized from total RNA using a reverse transcription kit (Takara). The primers used for the qPCR analysis of mRNA and miRNA levels are listed in Supporting Information, Table S1. The mRNA level of *act-1* was used as the internal standard. For the qPCR of the miRNA molecules *mir-84*, *mir-48*, and *mir-241* the reverse transcription primers used were mir-84-RT, mir-48-RT, and mir-241-RT, respectively. The complementary DNA products of all of the miRNAs contained a common sequence recognized by the mir-R primer. U18 snoRNA was used as the internal standard for miRNA quantification. qPCR was conducted on an ABI 7500 Fast real-time PCR system using a Takara real-time PCR kit (SYBR Premix Ex TaqTM II).

### Co-immunoprecipitation

Asynchronized worms were cultured on 100-mm, high-growth plates seeded with OP50 bacteria. Worms were incubated at 33° for 1.5 hr to induce the expression of HA-tagged DAF-12 from a transgene driven by a heat shock promoter. Worms were then incubated at 20° for 2.5 hr and subsequently harvested. Examination of the worms under a fluorescence microscope revealed that DAF-16::GFP accumulated in the nucleus at harvest. After washing with M9 buffer, 200 μL of packed worms were mixed with 200 μL of 2× lysis buffer [40 mM Tris pH 8.0, 200 mM NaCl, 0.2% NP-40, 20% glycerol, 2× Protease Inhibitor Cocktail (EDTA free; Roche)] and 800 μL of 0.5-mm diameter glass beads. The mixture was lysed using a FastPrep-24 (MP Biomedicals) homogenizer with 3 pulses (6.0 m/s, 20 sec/pulse); pulses were separated by 5-min intervals during which the samples were kept on ice. The lysate was separated from the glass beads and centrifuged at 15,700 × *g* for 30 min to remove worm debris. The lysate was incubated at 4° with anti-HA or anti-GFP agarose for 2 hr and washed 3 times with 1× lysis buffer, 5 min per wash, and then boiled in 2× sodium dodecyl sulfate loading buffer for Western blot analysis. The antibodies used were monoclonal anti-HA agarose (Sigma-Aldrich), anti-GFP agarose (made in-house), rabbit anti-HA (made in-house), mouse anti-GFP (Roche), goat anti-mouse IgG HRP (Jackson ImmunoResearch), and goat anti-rabbit IgG HRP (BaiHuiZhongYuan).

## Results

### The endogenous DA concentration of the long-lived *glp-1* mutant is not greater than that of the WT

**Figure 1:**
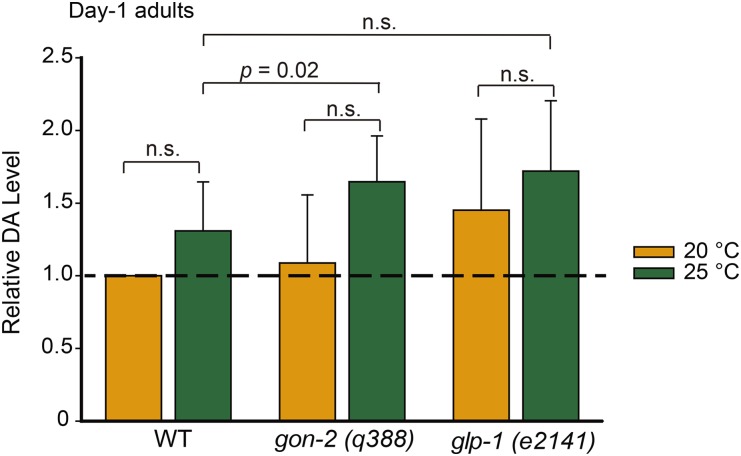
Endogenous dafachronic acid (DA) concentrations in the wild type (WT), *gon-2(q388)*, and *glp-1(e2141)* day-1 adults cultured at 20° or 25°. The DA concentrations were averaged over three independent experiments and normalized to level of the WT samples cultured at 20°. The absolute quantitation values are listed in Table S2. Error bars indicate the SD. Student’s *t*-tests were used for statistical analysis. n.s., not significant (*P* > 0.05).

We recently developed an accurate and sensitive LC-MS method to quantify endogenous DA levels by using deuterium-labeled DA as an internal reference (Li *et al.* 2013). Using this method, we measured the DA concentrations in the *glp-1(e2141ts)* and *gon-2(q388ts)* mutants. Different from the *glp-1(e2141)* mutant, which has an intact somatic gonad, the *gon-2(q388)* mutant lacks both the germ cells and the somatic gonadal cells when grown at 25° and is not long-lived. At 25°, we expected to find that the DA concentration in the *glp-1(e2141)* mutant would be greater than that of the WT, whereas the DA concentration in the *gon-2(q388)* mutant would be similar to that of the WT. However, we did not detect any significant differences between the WT and the *glp-1 (e2141)* mutant on adult day 1 (Figure 1 and Table S2) or at the L2/L3 larval stage (Figure S1 and Table S2), and the DA concentration in the *gon-2* mutant was slightly greater than that in the WT at 25° (*P* = 0.02, Student’s *t*-test). Moreover, *glp-1 (e2141)* mutant animals cultured at 20° and those cultured at 25° had similar endogenous DA concentrations.

We also measured the DA concentration in an additional *glp-1 (e2144)* mutant; this mutant shares the same temperature-sensitive sterile phenotype with *e2141*. As with the mutant of the *e2141* allele, the *glp-1(e2144)* mutant had an adult lifespan 16.7% longer than WT when cultured at 25° from the L1 stage (survival curve not shown). At the restrictive temperature of 25°, we did not observe a significant increase in DA concentration in L2/L3 larvae or in day-1 adults compared with the WT control (Figure S2 and Table S3). These results show that loss of *glp-1* activity has little effect on DA concentration, a supposition that runs counter to the commonly held idea that loss of *glp-1* activity prolongs lifespan by increasing the concentration of DA. Given that increased longevity of the *glp-1* mutant does require DA signaling (Shen *et al.* 2012; Yamawaki *et al.* 2010), we propose an alternative hypothesis in which the *glp-1* mutant has an enhanced response to DA; that is, the loss of germ cells increases sensitivity to DA but does not alter the endogenous DA concentration.

### Regulation of lifespan through altered sensitivity to DA

**Figure 2:**
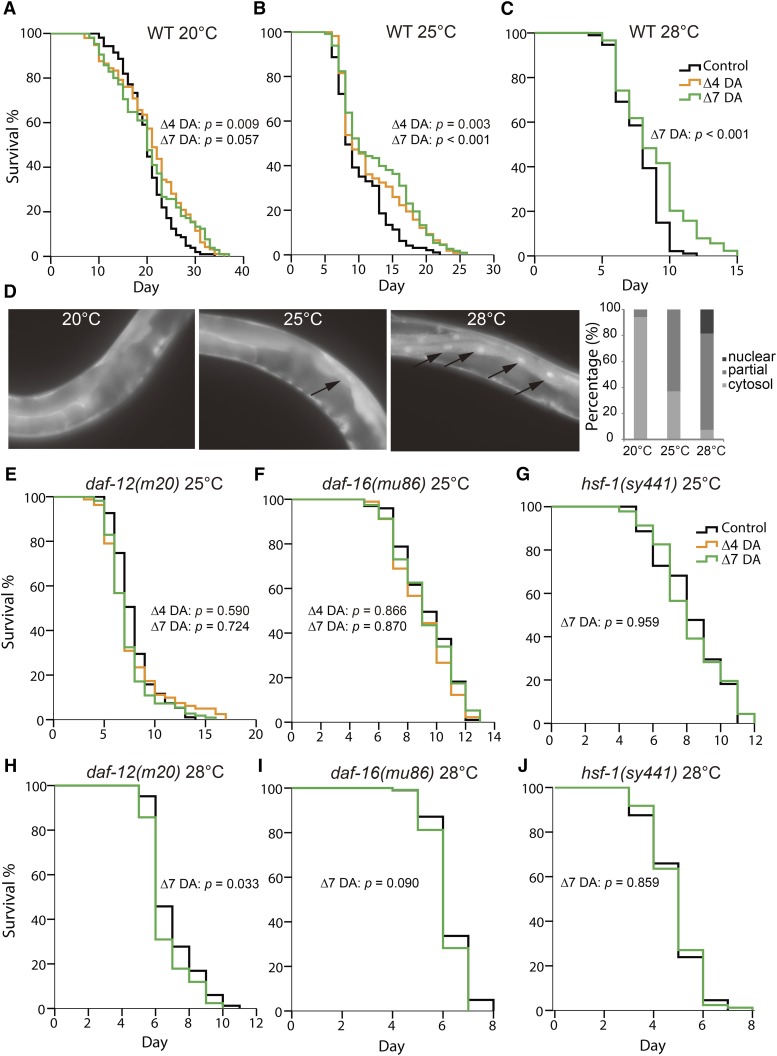
Exogenous dafachronic acid (DA) extended the lifespan of wild type (WT) worms at elevated temperatures in a *daf-12*-, *daf-16*-, and *hsf-1*-dependent manner. (A−C) Exogenous DA supplemented only during adulthood extended the lifespan of WT worms cultured at 25° (B) and 28° (C), but not at 20° (A). (D) WT day-1 adults exhibited weak or strong nuclear accumulation of DAF-16::GFP (indicated by arrows) 12 hr after a temperature shift from 20° to 25° or 28°. (E−J) The lifespan extension effect of DA at 25° and 28° required *daf-12* (E, H), *daf-16* (F, I), and *hsf-1* (G, J). Δ4-DA was not tested in the lifespan assays at 28° nor in the lifespan assay of the *hsf-1* mutant at 25°.

We further explored the possibility that lifespan may be modulated by sensitivity to DA. We fed WT worms DA during adulthood and found that DA extended the lifespans of worms grown at 25° and 28°, but DA had very little effect on the lifespans of worms grown at 20° (Figure 2, A−C and Table S4). Two DA isoforms 25*S*-Δ4-DA and 25*S*-Δ7-DA (Mahanti *et al.* 2014; Motola *et al.* 2006) were tested; both yielded similar results. Most of the subsequent experiments in this study were performed with the 25*S*-Δ7-DA, the more potent isoform (Mahanti *et al.* 2014; Motola *et al.* 2006).

The finding that the application of DA extends lifespans for worms grown at 25° and 28°, but not 20°, can be explained by either DA deficiency or increased sensitivity to DA at greater temperatures. Multiple independent quantitation experiments showed that there was no significant difference in the DA concentration between the worms grown at 25° and those grown at 20° on adult day 1 (Figure 1 and Figure S2C). The DA concentration might be greater in the L2/L3 larvae grown at 25° than those grown at 20°, but the difference was small and not always significant (Figure S1 and Figure S2D). Therefore, we propose that the worms grown at 25° are more sensitive to the longevity-promoting effect(s) of DA.

It is intriguing that DA hypersensitivity resulting from high temperatures is accompanied by the nuclear accumulation of DAF-16 (Figure 2D) (Lin *et al.* 2001); this observation suggests that some form of DAF-16 activation is involved. The lifespan extension effect of DA on WT worms grown at 25° and 28° was found to be completely dependent on *daf-12*, *daf-16*, and *hsf-1* (Figure 2, E−J and Table S4). This resembles the longevity of the *glp-1* mutant, which is also dependent on *daf-12* (Arantes-Oliveira *et al.* 2002), *daf-16* (Arantes-Oliveira *et al.* 2002), and *hsf-1* (Hansen *et al.* 2005).

### Daf-d mutant larvae have decreased DA levels

**Figure 3:**
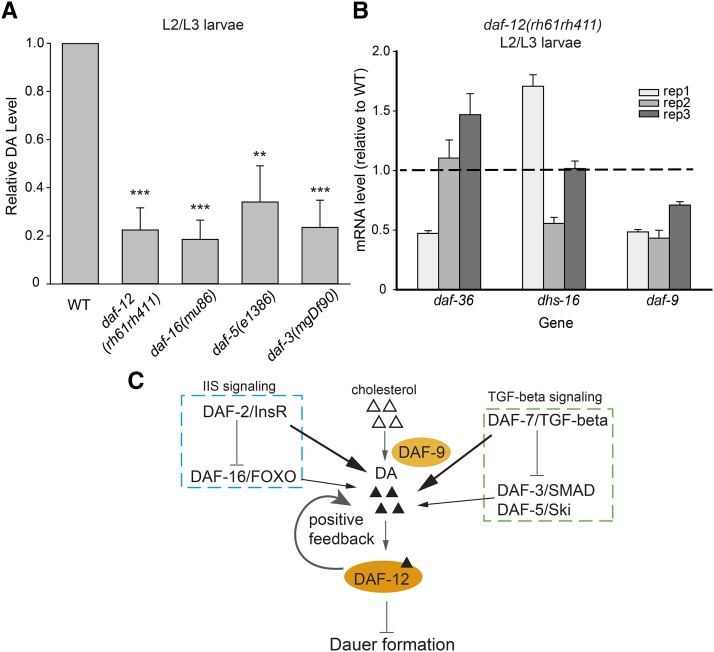
Dafachronic acid (DA) concentrations in the Daf-d mutants. (A) Relative DA levels in the Dauer formation-defective Daf-d mutant larvae cultured at 25°, expressed as averages ± SDs (n = 3). The values are listed in Table S5. Student’s *t*-tests were used for statistical analysis. ***P < 0.001, **P < 0.01, *P < 0.05. (B) mRNA transcripts of *daf-9*, but not *daf-36* or *dhs-16*, were significantly reduced in the *daf-12*−null mutant (*P* = 0.02 for *daf-9* mRNA). Results of three independent biological replicates are shown. (C) Model of DA regulation in dauer formation.

In addition to its role in aging, DA signaling is involved in the regulation of dauer formation. We previously quantified DA levels in several Daf-c mutants and found that compared with the WT L2/L3 control, these mutants all had extremely low DA concentrations before they entered the dauer stage (Li *et al.* 2013). The Daf-c phenotype of these mutants is caused by DA deficiency and can be rescued by supplying exogenous DA. As such, it might be expected that animals with the opposite phenotype, that is, the Daf-d mutants, may have high levels of DA at the L2/L3 stage; this supposition has not been verified to date. In this study, we found that the DA concentration was only 19–34% of the WT level in each of the four Daf-d mutants assayed—*daf-12(rh61rh411)*, *daf-16(mu86)*, *daf-5(e1386)*, and *daf-3(mgDf90)* (Figure 3A and Table S5).

It is intriguing that DA deficiency did not drive these Daf-d mutants into the dauer state. In the case of the *daf-12* mutant, it is understandable because the dauer program is turned on by ligand-free DAF-12 together with its corepressor DIN-1 (Ludewig *et al.* 2004), which competes with DA for DAF-12 binding (Motola *et al.* 2006). In the absence of DAF-12, DIN-1 itself cannot induce dauer formation. For the other three mutants, there must be a different explanation. One possibility is that the expression level of *daf-12* or *din-1*, or both, is reduced. However, this notion was not supported by the qPCR results (Figure S3). Another possibility is that the DA level is not sufficiently low. We previously determined that the DA levels in the pre-dauer Daf-c worms [*daf-2(e1370)*, *daf-2(e1368)*, *daf-7(ok3125)*, *daf-11(ks67)*] were each less than 15% of the level of the WT control (Li *et al.* 2013). Given that the DA levels in the Daf-d mutants were 19–34% of those in the WT, we propose that perhaps the critical threshold is somewhere between 15% and 19%. Supporting the idea that DA concentration is not sufficiently low to drive dauer formation in Daf-d mutants, the *daf-9(e1406)*; *daf-16(m26)* double mutant has the Daf-c phenotype (Jia *et al.* 2002). *daf-9* encodes a key enzyme in DA synthesis and *daf-9* null animals have no detectable DA.

Curiously, despite the reduced DA levels in the *daf-16*, *daf-3*, and *daf-5* mutants, the activity of DAF-12 seems largely unaffected, as shown by the WT-like expression levels of the *let-7* family miRNAs (Figure S4); these are known to be transcribed by activated, DA-bound DAF-12 (Bethke *et al.* 2009). Because *daf-16*, *daf-3*, and *daf-5* encode transcription factors, we asked whether they positively regulate the genes required for DA synthesis. However, the expression levels of the genes encoding the enzymes of the DA synthesis pathway (*daf-9*, *daf-36*, and *dhs-16*) (Gerisch *et al.* 2001; Jia *et al.* 2002; Rottiers *et al.* 2006; Wollam *et al.* 2012) were similar in the WT and the *daf-16*, *daf-3*, and *daf-5* mutants (Figure S5).

### Positive feedback between DA production and DAF-12 activity

A positive feedback mechanism has been proposed based on the observation that the expression of a DAF-9::GFP fusion protein is reduced in the hypodermis of the *daf-12(rh61rh411)*-null mutant (Gerisch and Antebi 2004; Schaedel *et al.* 2012). This assertion is supported by our quantitation results showing that the DA concentration was indeed reduced by almost 80% in the *daf-12*−null mutant (Figure 3A). We further verified that the *daf-9* mRNA level was reduced by approximately 50% in the *daf-12* mutant (Figure 3B). All of these results support the hypothesis that there is a positive feedback loop between DA production and DAF-12 activity.

## Discussion

The observation that DA/DAF-12 signaling can increase lifespan under conditions that do not alter endogenous DA levels (for example, *glp-1* mutation and growth at 25°) led us to the hypothesis that these conditions might induce a prerequisite change that enables DA to extend lifespan, which we refer to as enhanced sensitivity to DA. Consistent with this idea, it has been shown that DA supplemented in the culture medium can fully rescue the lifespan phenotype of a weak, short-lived *daf-9(rh50)* mutant to the WT level at 22.5°, but not at 20° (Gerisch *et al.* 2007). It is worth noting that DAF-16 accumulates in the nucleus in both the *glp-1* mutant and the WT worms at 25° and above (Lin *et al.* 2001), which suggests a potential link between DAF-16 activity and the mechanism that defines sensitivity to DA in *C. elegans*. *daf-9* and *daf-12* are partially required for the nuclear accumulation of DAF-16 in worms lacking a germline (Berman and Kenyon 2006; Gerisch *et al.* 2007). However, whether DAF-12 and DAF-16 regulate common downstream targets remains a controversial topic. In worms deprived of germ cells, the expression of *sod-3*, a DAF-16 target gene, is largely independent of *daf-12* (Yamawaki *et al.* 2010), whereas in the *hsd-1*; *akt-1* double-mutant background, *daf-12* and *daf-16* are both required for the expression of the *sod-3* gene (Dumas *et al.* 2010). Also, we were unable to detect any physical interaction between HA-tagged DAF-12 expressed under a heat shock promoter and GFP-tagged DAF-16 expressed under its own promoter (Figure S6). This finding does not support an interaction between DAF-16 and DA/DAF-12 but does not rule out this possibility, either. Further examination of the DAF-16 and DAF-12 dependent transcriptional profiles would help test the hypothesis that DAF-12 acts as a co-activator of DAF-16 under certain conditions.

In 2012, a fivefold increase of Δ7-DA was reported in the long-lived *glp-1 (e2141)* mutant relative to the WT (Shen *et al.* 2012). No such increase was detected in this study. Using our quantitation method, we measured a slight increase in the DA concentration of the *glp-1 (e2141)* mutant, but this increase was not statistically significant. This discrepancy may have to do with differences in the bioanalytical methods used for DA quantification. The method used in this study was described in full in 2013 (Li *et al.* 2013). In contrast, some of the details including the lower limit of quantification, the linear range of quantification, the accuracy, and the relative standard deviation of the method used in the 2012 study are not available. Therefore, it is difficult to compare the two methods to identify possible causes for the differences in quantitation results. Based on the information that is available, we speculate that one important factor may be the internal standard used for DA quantification. The ideal internal standard should be one that is not present in *C. elegans* and one that possesses the same chemical and physical properties as the target compound but has a different mass. We have evaluated the performance of various natural steroid compounds including cholic acid, progesterone, and testosterone as candidate internal standards, but did not obtain satisfactory results (not shown). Only with [d_3_]-DA, which fits all the criteria of an ideal internal standard, did we obtain reproducible results that made biological sense. 5β-cholanic acid, which was used in 2012 (Shen *et al.* 2012), is probably not the best internal standard. Another factor might be analyte selectivity: the method used in 2012 may be selective toward Δ7-DA, while our method measures the total amount of Δ4- and Δ7-DA because it cannot discriminate these two forms. It is possible that the level of Δ7-DA is increased in *glp-1* mutant worms, but the level of Δ4-DA is decreased and could offset the increase of Δ7-DA; this scenario would result in little net change in the total amount of Δ4- and Δ7-DA. However, according to another method that enables separate quantification of Δ4-DA and Δ7-DA, the amount of Δ4-DA is approximately 30% of that of Δ7-DA in the WT (Witting *et al.* 2015). Therefore, if the total amount of Δ4- and Δ7-DA remained the same, the maximal increase of Δ7-DA would be approximately 30%, far lower than the fivefold increase reported in 2012. Moreover, an nuclear magnetic resonance study detected Δ7-DA, but not Δ4-DA, in *C. elegans* (Mahanti *et al.* 2014). It is clear that these results cannot all be correct at the same time, but it is difficult to solve the controversy using the methods that have been described to date. Solving this controversy would require highly sensitive analytical methods that can separately quantify Δ4-DA and Δ7-DA, preferably using stable isotope-labeled Δ4-DA and Δ7-DA as internal standards.

Recently, several related but distinct compounds, including 25*S*-Δ1,7-DA and 3-alpha-OH-Δ7-DA, have been identified as ligands of DAF-12 (Mahanti *et al.* 2014). Our DA bioanalytical method cannot measure the concentrations of these ligands. It is possible that the levels of these compounds may differ between the *glp-1* mutants and the WT.

With respect to dauer formation, we found that the total concentrations of Δ4- and Δ7-DA were reduced in both the Daf-c (4–15% of WT) (Li *et al.* 2013) and Daf-d mutants (19–34% of WT), indicating the complexity of DA regulation. Our finding of a low DA concentration in the *daf-12*−null mutant is consistent with a previous observation that loss of *daf-12* activity decreases the expression of a *daf-9*::*GFP* transgene (Gerisch and Antebi 2004), and these results strongly support the existence of positive feedback between DA production and DAF-12 activity. A recent study found that positive feedback was induced when the DA level reached a certain threshold (Schaedel *et al.* 2012). Based on our results, we suggest that in the Daf-c mutants, the DA concentration (including Δ4-DA, Δ7-DA, 25*S*-Δ1,7-DA, and 3-alpha-OH-Δ7-DA) is below this hypothetical critical threshold, so no positive feedback is induced and the worms arrest as dauers. In contrast, the *daf-16*, *daf-3*, and *daf-5* mutants do not form dauers and express *daf-9* mRNA and the *let-7* family miRNAs at near WT levels, despite low concentrations of Δ4- and Δ7-DA. We therefore reason that positive feedback is partially induced in these Daf-d mutants; either the DA concentration threshold is between 15% and 19% of the WT level, or these Daf-d mutants have a sufficient amount of 25*S*-Δ1,7-DA or 3-alpha-OH-Δ7-DA, or both, to make up for the scarcity of Δ4- and Δ7-DA. These results also show that, for both insulin signaling and TGF-β signaling, the upstream components both positively and negatively regulate DA (Figure 3C). Taking insulin signaling as an example, the loss of either *daf-2* or *daf-16* activity results in DA deficiency, suggesting that both of these positively regulate DA levels. Yet, it is well established that DAF-2 inhibits DAF-16 and that DAF-16 is activated in *daf-2* mutants, so, if DAF-16 mediates all signaling from DAF-2, *daf-2* mutants should have a surplus of DA, instead of DA deficiency. Therefore, there must be a branch of signaling from DAF-2 that promotes DA production or inhibits DA degradation, or both, that acts independently of DAF-16. Likewise, there is most likely a branch of DAF-7 signaling that positively regulates DA independently of DAF-3 and DAF-5 (Figure 3C).
